# Prevalence and pattern of traumatic limb amputations in female population of Bhimber District, Azad Jammu and Kashmir, Pakistan

**DOI:** 10.12669/pjms.311.6423

**Published:** 2015

**Authors:** Nazish Jabeen, Sajid Malik

**Affiliations:** 1 Nazish Jabeen, Human Genetics Program, Department of Animal Sciences, Faculty of Biological Sciences, Quaid-i-Azam University, 45320 Islamabad; 2 Sajid Malik, Human Genetics Program, Department of Animal Sciences, Faculty of Biological Sciences, Quaid-i-Azam University, 45320 Islamabad

**Keywords:** Traumatic limb amputations, Women health, Epidemiology, Prevalence, Pakistani subjects, Bhimber population

## Abstract

**Objective:**

Traumatic limb amputations (TLA) are structural defects that cause mild-to-severe disabilities and have significant impact on the quality of life of subjects. A recent epidemiological study in Bhimber District, AJK, Pakistan, revealed that TLA had a very high incidence among the young/adult females. The present study aimed at determining the prevalence, pattern, causes and risk factors associated with TLA in that female sample.

**Methods:**

In a prospective door-to-door survey, 1731 females were randomly enrolled, and clinico-etiological investigations on 19 subjects with TLA were carried out in a follow-up study. Affected subjects were physically examined and phenotypic detail and restriction to normal function were documented.

**Results:**

There were 19 subjects with TLA, hence the prevalence was estimated to be 10.98/1,000 (0.011%; CI=0.0061-0.0159). TLA mostly involved the upper limbs and right hand. Transphalangeal amputations were most frequent, followed by involvements of middle/upper arm and leg segments. These analyses further revealed that agriculture tools were the leading cause of trauma. TLA were witnessed to be higher in subjects originating from Samahni tehsil (OR:2.71), rural areas (OR:3.33), those who were illiterate (OR:2.65), married, spoke Pahari language (OR:1.73), those who fall in higher age category (OR:16.74), and had certain professions.

**Conclusion:**

Limb amputations had heavy bearings on the lives of subjects. Curiously, majority of these traumas could be avoided by adopting certain safety measures. Prevalence and risk estimate of TLA across various socio-demographic variables of Bhimber population would be useful in guiding further studies and the public health policy to mitigate the impact of these anomalies.

## INTRODUCTION

Traumatic limb amputations (TLA) are structural defects that cause mild-to-severe disabilities in men, women, and children in all countries. The accurate number of individuals who have had amputations globally is difficult to determine. Many societies particularly in the developing world do not have reliable data on number of subjects with amputations or their causes.^1^ Non-industrialized nations generally have elevated prevalence due to high incidences of wars, accidents, and less developed medical systems. There is no systematic documentation of subjects with limb amputations in Pakistan.

The primary causes of amputation are diseases (i.e., vascular diseases, diabetes, tumors and malignancies, infections), accidents/trauma, and congenital deformities.^1^^,^^2^ A country-wide study in Korea showed that the most common cause of amputation was trauma.^3^^,^^4^ Al-Turaiki and Al-Falahi observed that in Saudi Arabia during 1977-1990, 86.9% of the upper limb and 52.9% of lower limb amputations were due to trauma.^5^ In sub-Saharan Africa, tumors and trauma were observed to be the main causes.^6^ Traumatic amputations tend to occur in younger and active age.^1^^,^^3^ Compared to females, males have a significantly higher risk for trauma-related amputations.^7^

Several epidemiological studies have focused on morbidities in the Pakistani sub-populations.^8^^,^^9^ However, little is known about TLA. A recent study showed a high prevalence of TLA in the female population of Bhimber District, north-east of Pakistan.^10^ In current research, we carried out a follow-up clinico-epidemiological investigation to observe the prevalence, pattern, etiology and risk factors associated with TLA occurring in the Bhimber sample.

Bhimber District is the southernmost of the ten districts of Azad Jammu and Kashmir (AJK), Pakistan. It comprises three tehsils and 19 union councils. District’s population is 0.401 million individuals (2009 projections), and annual growth rate is estimated to be 2.6%. The primary languages are Punjabi and Pahari.^10^^,^^11^ The Bhimber population is characterized by rural community, extended household size (average 6.7), very high rate of consanguineous unions (62%), low socio-economics and about 60% literacy rate.^12^ The District has mainly an agro-based economy. Women in rural areas are actively involved in farming and cattle-work, and are therefore, on a high risk of injuries associated with agriculture tools/machinery.

## METHODS

***Methodology: ***The ascertainment of subjects with TLA was prospective to a population-based study conducted during 2010 in Bhimber District of Azad Jammu and Kashmir. The detailed study design, survey and sampling are reported in Jabeen and Malik.^10^ Briefly, 1,731 females of age 12-75 years were ascertained from 24 different sampling sites of three tehsils of Bhimber District. These females were approached through door-to-door surveys or in public places. The enrolled subjects were interviewed in face-to-face contacts and physically examined with the help of the resident doctors. In that sample, TLA were witnessed to have the highest representation among the non-communicable disorders, which warranted further study.^10^ For the current independent research, detailed epidemiological and clinico-etiological data on 19 subjects with TLA were collected. It was essentially a multi-step follow-up investigation carried out during 2012-2013. In the first step, an informed consent was obtained from each subject or her family/husband. In the second step, each subject was re-approached at her home and descriptive data were obtained. In the third step, subjects underwent a detailed physical and clinical examination with the help of local medical practitioners. Information was also acquired on medical help available at the time of accident, prosthetic management, and adaptation after the amputation/accident. All subjects consented to give clinical information; however, only eleven subjects consented to provide photographs. This study was approved by the ethical review committee of Quaid-i-Azam University, Islamabad (No.DAS/13, June 3, 2013).

The distribution of TLA was established with respect to the socio-demographic attributes of subjects. Prevalence of TLA was calculated in the total sample and was presented as per 1,000 individuals. Prevalences were also estimated for the individual socio-demographic categories. Confidence interval (CI) was measured from the proportion in a respective category. Odd ratios were calculated to estimate the risk relative to the socio-demographic categories.^13^

## RESULTS

Among the 1731 enrolled females, there were a total of 19 subjects with TLA which belonged to tehsils Samahni (n=9), Barnala (n=5), and Bhimber (n=5). Accordingly, the prevalence of subjects with TLA was calculated to be 10.98/1,000 (0.011%; CI=0.0061-0.0159) in the young/adult female population of Bhimber District.

***Pattern of TLA: ***The recruited subjects were observed to have a total of 20 amputations (Table-I). Upper limbs were most frequently affected than the lower limbs (18 vs. 2) (Fig.I). Within the upper limbs, the right arm was most commonly affected than the left arm (11 vs. 7). In majority of the amputations, phalanges were affected (n=16), followed by the middle/upper arm (trans-radial, trans-humeral/elbow; n=3), and upper leg (trans-femoral; n=1). With respect to the affected autopodal axis, majority of the amputations affected the mesoaxial axis (n=9), followed by postaxial (n=4) and preaxial involvements (n=4) (Fig-I). Depending upon the level of amputation, the cases were divided into three severity grades: mild, moderate and severe. In mild cases, only the terminal phalanx of a certain digit of hand/foot was omitted; in moderate cases, amputation resulted in loss of a complete digit; and in severe forms more than one limb segments or whole autopod was involved. Accordingly, most of the cases had milder nature (n=11), followed by moderate (n=5), and severe (n=4) amputations (Table-I) (Fig.IIA).

***Causes of TLA: ***Agriculture tools were the most common cause of TLA (n=9), followed by road accidents (n=3), domestic violence (n=3), fall injury (n=2), and firearms/mines (n=2) (Fig.IIB). Subjects were also inquired about the availability of medical help at the time of accident. Only six subjects witnessed that they were able to get medical treatment soon after the accident/amputation and were operated subsequently. Most of the subjects relied only on traditional healing methods and domestic tips for their recovery. However, none of the subjects was using any prosthetic device.

***Distribution of subjects with TLA in demographic variables: ***The subjects were ranging 24 to 66 years in age (mean: 43.3212.55). There was a high prevalence of cases from tehsil Samahni (prev.:17.54/1,000; OR:2.71). The high prevalence of TLA was associated with Pahari language (OR:1.73), married status (prev.:11.99/1,000), rural origin (OR:3.32), illiteracy (OR:2.56), and increasing age (Table-II). Risk was generally higher in women who were engaged in certain professions. Among the major castes (sample size >200), the highest prevalence was observed in Mirza, followed by Rajput and Gujjar (Table-II).

## DISCUSSION

TLA are one of the most common causes of permanent disabilities. In the United States, there are about 1.7 million individuals with limb loss.^14^ The effect of TLA could be physical, psychological and social. Adaption in all instances is not easy. Subjects with amputations experience a wide range of activity limitations and restrictions, including self-care, mobility and occupational activities.^1^^,^^3^ In many cases, prosthetic management and adequate treatment is not possible. TLA may also cause disfiguration of body image. These affect the ability of the affectee to return to work, maintain social relations, participate in leisure activity and be an active member of society.^3^^,^^5^

There is a dearth of knowledge about the limb amputations in Pakistan. This study presents the prevalence and dynamics of TLA in female population of Bhimber District, which appeared to carry a high burden of such defects. In the present sample, most of the amputations affected the upper limbs. This observation is consistent with a study carried out in US by Dillinghan et al., who observed that upper-limb amputations accounted for the 68.6% of all trauma-related amputations.^7^ On the other hand, lower limb amputation were more common in Korea (i.e., 69%) compared the upper limb.^4^ In most of the subjects presented in this cohort, amputations affected only the phalanges. This group of malformations was rather milder in nature and had minimal effect on daily activities of the subject. However, in the experience of Kim et al., amputations were of severe in nature more often and trans-tibial and trans-femoral amputations were common.^4^ In our subjects, the more severe cases of amputations resulted in restricted domestic activities and adversely affected their quality of life. In all the cases nonetheless, amputations resulted in disfiguration of limbs. Detailed interviews revealed that majority of the subjects (n=11/19) believed that amputations were causes of stigma and restricted their social life.

The present study revealed that emergency help at the time of accident/trauma was not available to most of the subjects (n=13/19; 68%). In the rural and low socioeconomic backgrounds, people usually have poor medical help-seeking-behavior. They rely on traditional healers and domestic tips and do not visit the qualified doctor/hospital until the situation worsens and becomes life threatening.^15^ Thus, none of the subject had access to prosthetic aids, mainly due to poverty and lack of specialized hospitals in their remote neighborhoods.

The etiology of limb amputation varies from globally. In developed countries, industrial accidents, motor vehicle accidents or farming accidents, are the major causes of trauma. In certain developing nations however, infected insect, animal and human bites and other wounds are the main causes of limb amputation. Inappropriate use of traditional medicines for remedy may also increase infections that can lead to amputation.^1^ In the rural areas of Bhimber District, similar to other regions of Pakistan, women traditionally work in the fields and significantly contribute in subsistence farming and cattle-work. Conventional and non-automated tools employed in the fields/cutting fodder, put the women at a higher risk of accidents. Kohler et al. also observed that the leading cause of trauma-related amputation were the injuries involving machinery, powered tools, firearms, and road accidents.^3^ In the present study, the recruited subjects submitted during the interviews that accidents occurred due to poor orientation and improper use of agriculture tools, non-compliance with the safety measures, lack of emergency medical facilities, and poor medical help-seeking-behavior. In a study in Burma, Hla^16^ observed that trauma was the most common cause in both upper and lower limb amputations. The authors further showed that trauma accounted for 87% and 47% of upper and lower limbs amputations, respectively.^16^

Besides road-side accidents, domestic violence also appeared as the minor source of TLA in the present study. Domestic violence is not much debated in Pakistan, but is not rare particularly in the rural, illiterate and low socio-economic communities of the country. The empirical data on this issue is scarce.^17^ Another cause of TLA was firearms/mines, which appear to be a constant risk factor for communities residing near the line-of-control. The geopolitical situation at the political boundaries of AJK (Pakistan) and Indian held Kashmir remains uncertain and women and children are the most usual victims of cross-border conflicts. Different studies have highlighted the impacts of war-situation on the health of Kashmiri women residing close to line-of-control.^18^

**Table-I:** Various attributes of subjects with TLA

**Subject #**	**Affected limb (n=20)**	**Affected limb segment**	**Amputation point**	**Severity**	**Causes (accident/trauma type)**	**Medical help available**
1	RL, LA	Knee, 1^st^ digit	Trans-tibial; trans-phalangeal	Severe; mild	Road accident	Yes
2	RA	3rd digit	Trans-phalangeal	Mild	Road accident	
3	RA	3rd digit	Trans-phalangeal	Mild	Agri. tool	
4	RA	3rd digit	Trans-phalangeal	Moderate	Domestic violence	Yes
5	RA	Arm	Trans-radial	Severe	Fall injury	
6	RA	Middle arm/elbow	Trans-radial/elbow	Severe	Fall injury	Yes
7	LA	3rd digit	Trans-phalangeal	Mild	Agri. tool/sickle	
8	RA	1st digit	Trans-phalangeal	Mild	Agri. tool	
9	LA	4th digit	Trans-phalangeal	Mild	Domestic violence	
10	RA	5th digit	Trans-phalangeal	Severe	Domestic violence	Yes
11	RA	5th digit	Trans-phalangeal	Severe	Firearm/mines	
12	LA	3rd digit	Trans-phalangeal	Mild	Agri. tool/sickle	
13	RA	Middle arm	Trans-humeral/elbow*	Severe	Firearm	Yes
14	LA	5th digit	Trans-phalangeal	Moderate	Agri. tool/cotton machine	Yes
15	LA	5th digit	Trans-phalangeal	Mild	Agri. tool	
16	LL	4th toe	Trans-phalangeal	Mild	Road accident	
17	RA	1^st^ digit	Trans-phalangeal	Mild	Agri. tool	
18	RA	2nd digit	Trans-phalangeal	Mild	Agri. tool/grass cutter	
19	LA	1st, 3rd digit	Trans-phalangeal	Moderate	Agri. tool/grass cutter	

RA=right arm; RL=right leg; LA=left arm; LL=left leg; Agri.=Agriculture;

* limb is not amputated but hanging as nonfunctional organ

**Table-II:** Prevalence, proportions and ODD ratios of TLA across demographic differentials of recruited sample from Bhimber.

Demographic variable	Normal subjects	Amputee cases	Prevalence/ 1,000	Proportion	95% CI	ODD Ratio
*Tehsils*						
Bhimber	759	5	6.54	0.0065	0.0008-0.0123	Reference
Barnala	449	5	11.01	0.0110	0.0014-0.0206	1.690
Samahni	504	9	17.54	0.0175	0.0062-0.0289	2.710
Total	1712	19	10.98	0.0110	0.0061-0.0159	
*Mother tongue*						
Punjabi	1205	11	9.05	0.0090	0.0037-0.0144	Reference
Pahari	507	8	15.53	0.0155	0.0049-0.0262	1.728
*Marital status*						
Married	1565	19	11.99	0.0120	0.0066-0.0174	-
Single	147	0	0.00	0.0000	-	-
*Origin*						
Rural	1317	18	13.48	0.0135	0.0073-0.0197	3.325
Per-urban	152	0	0.00	0.0000	0.0000-0.0000	-
Urban	243	1	4.10	0.0041	-0.0039-0.0121	Reference
*Education* *						
Illiterate	518	10	18.94	0.0189	0.0073-0.0306	2.560
Literate	1194	9	7.48	0.0075	0.0026-0.0124	Reference
*Age groups (Yrs)* *						
Up-to 30	831	2	2.40	0.0024	-0.0009-0.0057	Reference
31-40	487	8	16.16	0.0162	0.0051-0.0273	6.828
41-50	245	3	12.10	0.0121	-0.0015-0.0257	5.089
Above 50	149	6	38.71	0.0387	0.0083-0.0691	16.737
*Occupation*						
House-wife	1391	16	11.37	0.0114	0.0058-0.0169	Reference
House-wife/cattle-work	144	2	13.70	0.0137	-0.0052-0.0326	1.208
Teacher	14	1	66.67	0.0667	-0.0596-0.1929	6.211
Student/other profession	163	0	0.00	0.0000	-	-
*Caste system*						
Jatt	742	5	6.69	0.0067	0.0008-0.0125	Reference
Rajput	318	3	9.35	0.0093	-0.0012-0.0199	1.400
Gujjar	215	2	9.22	0.0092	-0.0035-0.0219	1.380
Mirza	207	4	18.96	0.0190	0.0006-0.0374	2.867
Mughal	57	1	17.24	0.0172	-0.0163-0.0507	2.603
Malik	56	1	17.54	0.0175	-0.0165-0.0516	2.649
Butt	34	2	55.56	0.0556	-0.0193-0.1304	8.728
Syed	35	1	27.78	0.0278	-0.0259-0.0815	4.239
Others	48	0	0.00	0.0000	-	-
Total	1712	19	10.98	0.0110	0.0061-0.0159	

* Chi^2^ distribution was statistically significant. (Categories with nil values were not utilized in calculations).

**Fig-I:**
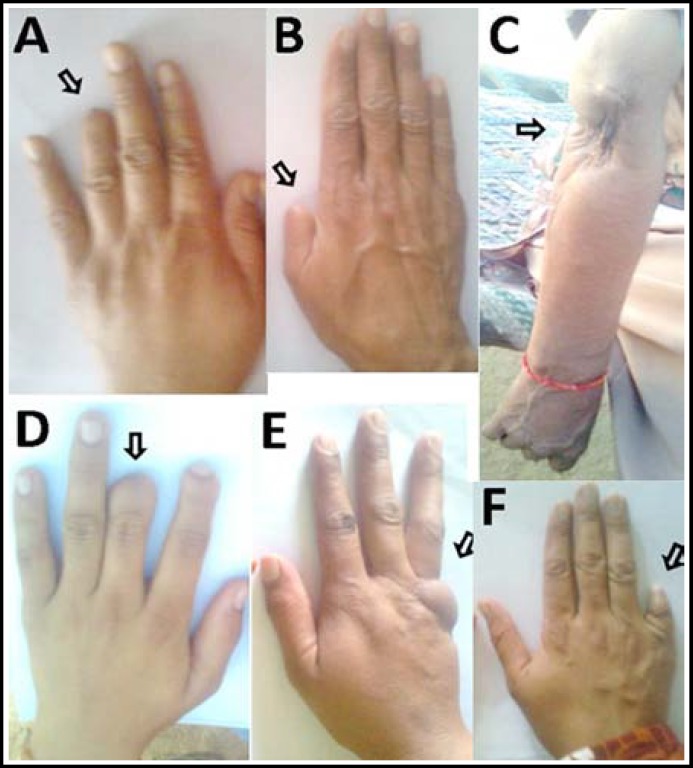
**A,B,D.** Terminal phalangeal amputations of 4^th^, 1^st^ and 3^rd^ digits in subjects# 9, 8 and 12, respectively. **C.** Firearm associated trauma in subject# 13 resulting in non-functional passively hanging arm which also had bluish appearance. **E. **Postaxial amputation of last digit in subject# 11. Post-traumatic healing and edema is visible. **F.** Postaxial amputation at the level of proximal phalange in subject# 10.

**Fig-II:**
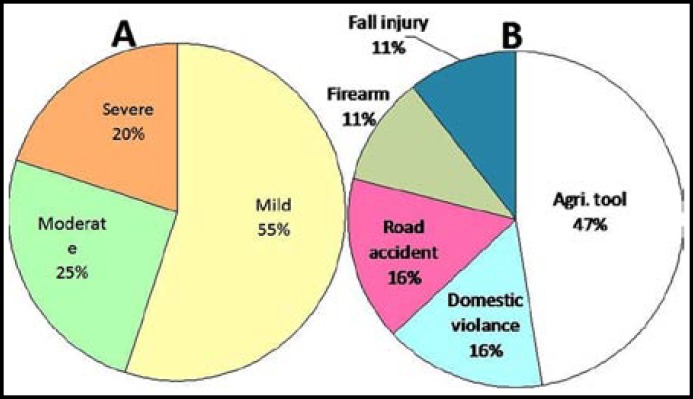
**A.** Pie chart depicting the percentage of severity grades of TLA. **B.** Pie chart depicting the causes of TLA.

Certain safety measures could potentially decrease the incidence of TLA in Bhimber population. The importance of safety measures and the proper use of tools/machinery should be strongly emphasized. Accessibility to the health facility could have had decreased the impact of trauma/accidents. The basic health units in the rural areas of Pakistan are poorly equipped and usually lack the facilities and capacity to handle emergency situations. Nonetheless, in order to fully appreciate the impact of TLA it would be worthwhile to explore the pattern of such malformations in various population strata of all districts of AJK, in a more comprehensive prospective study.

## CONCLUSION

This study presents important empirical data on the prevalence of TLA in the female population of Bhimber. Detailed presentation of subjects with TLA across key socio-demographic variables helps understand the distribution and risks associated with TLA. This study sets a baseline to carry out further studies on the same lines. These data could be highly valuable in guiding the public health policies in order to launch awareness and intervention programs, and to mitigate the impact of TLA in this population.

## Notes

***Source of funding:*** HEC-Pakistan and PSF-Islamabad.
